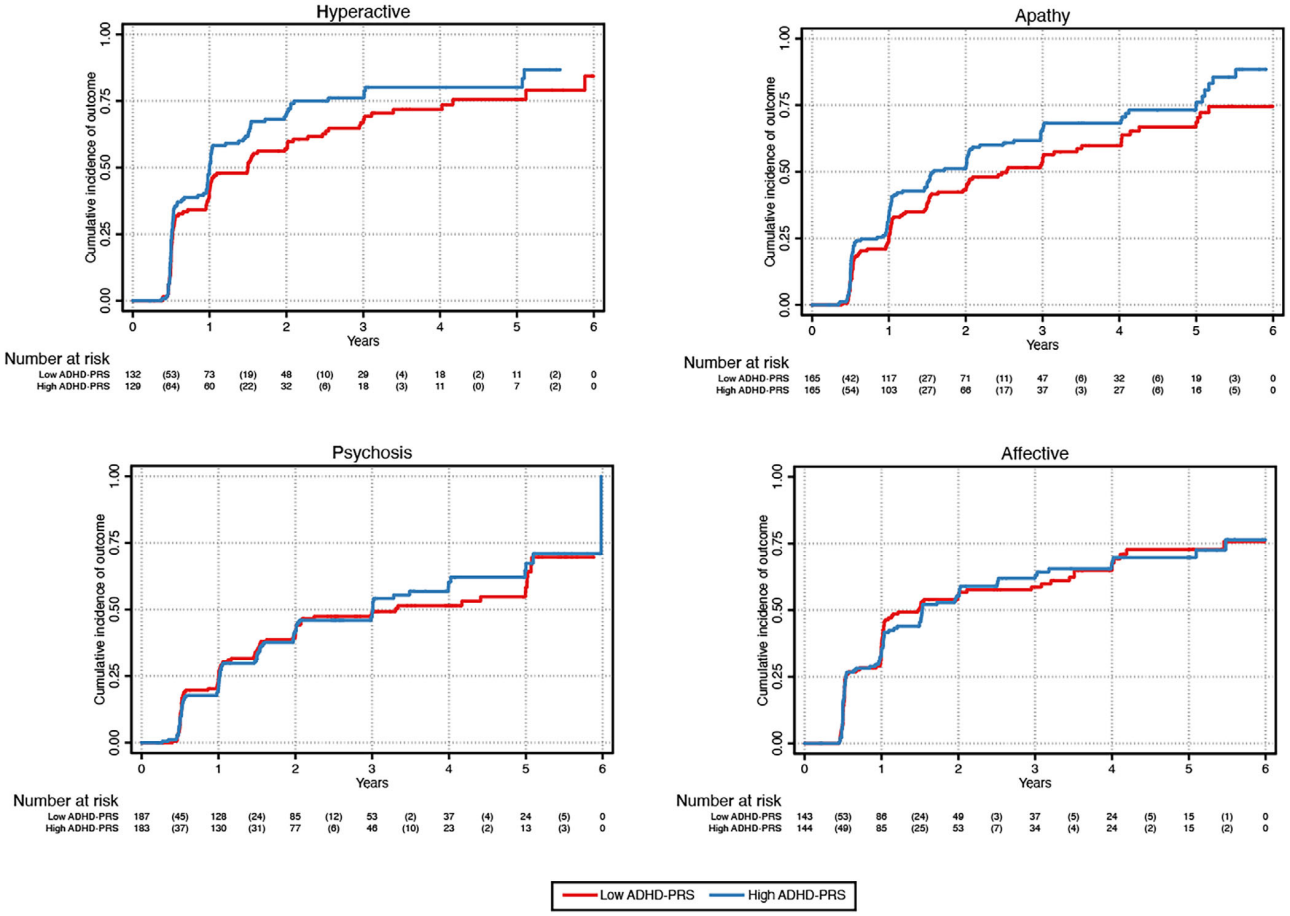# Attention‐deficit/hyperactivity disorder impacts the development of neuropsychiatric symptoms in Alzheimer’s disease

**DOI:** 10.1002/alz.089857

**Published:** 2025-01-03

**Authors:** Douglas Teixeira Leffa, Bruna Bellaver, Guilherme Povala, Pamela C. L. Ferreira, João Pedro Ferrari‐Souza, Firoza Z Lussier, Carolina Soares, Hussein Zalzale, Guilherme Bauer‐Negrini, Sarah Abbas, Thomas K Karikari, Luis Augusto Rohde, Brooke Molina, Tharick Ali Pascoal

**Affiliations:** ^1^ University of Pittsburgh, Pittsburgh, PA USA; ^2^ Universidade Federal do Rio Grande do Sul, Porto Alegre, Rio Grande do Sul Brazil; ^3^ Universidade Federal do Rio Grande do Sul, Porto Alegre Brazil

## Abstract

**Background:**

Recent studies have shown that patients with attention‐deficit/hyperactivity disorder (ADHD) are more likely to be diagnosed with mild cognitive impairment (MCI) and Alzheimer’s Disease (AD). Increased genetic risk for ADHD, measured with ADHD polygenic risk scores (ADHD‐PRS), was associated with a more severe AD presentation, including worse cognitive function and higher tau pathology. Neuropsychiatric symptoms (NPSs) are common in AD and are hypothesized to occur with disease progression. It is unclear whether ADHD predispose individuals to the development of NPSs.

**Method:**

We used data from the Alzheimer’s Disease Neuroimaging Initiative (ADNI) to investigate the associations between ADHD‐PRS and the development of NPSs in cognitively impaired (CI, including MCI and AD) subjects who had brain amyloidosis at baseline. NPSs were assessed with the NPI or NPI‐Q and divided into four syndromes: hyperactive (agitation, disinhibition, irritability, and aberrant motor behavior), psychosis (delusions, hallucinations, and night‐time behavior disturbances), apathy (apathy and appetite and eating abnormalities), and affective (depression and anxiety). Additionally, we tested the specificity of our findings by analyzing the association between the genetic risk for depression, bipolar disorder (BD), schizophrenia, and autism spectrum disorder (ASD) with the development of NPSs. Data were represented with Kaplan‐Meier survival curves, and analyses were performed with Cox proportional hazards regression models controlled by age, sex, and ancestry.

**Result:**

We evaluated data from 852 participants (636 MCI [74.1%], 350 women [40.8%], mean [SD] age of 73.4 [7.3] years). ADHD‐PRS was associated with a higher risk of developing hyperactive (HR 1.41; 95% CI 1.04‐1.89) and apathy (HR 1.34; 95% CI 1.01‐1.77) symptoms, but not psychosis (HR 1.04; 95% CI 0.78‐1.41) or affective (HR 0.97; 95% CI 0.72‐1.32) symptoms. There was no association between the genetic risk for depression, BP, schizophrenia, or ASD with any neuropsychiatric syndrome.

**Conclusion:**

Findings indicate that a higher genetic risk for ADHD may be a risk factor for the development of hyperactive and apathy symptoms in CI individuals. These effects were specific for ADHD and were not observed for other prevalent psychiatric disorders. Clinically, our results contribute to the literature that highlights the need for tailored assessments for CI individuals with ADHD histories.